# Current Application and Future Prospects of Artificial Intelligence in Healthcare and Medical Education: A Review of Literature

**DOI:** 10.7759/cureus.77313

**Published:** 2025-01-12

**Authors:** Girish Joseph, Neena Bhatti, Rithik Mittal, Arun Bhatti

**Affiliations:** 1 Pharmacology, Christian Medical College & Hospital, Ludhiana, IND; 2 Neurosciences, Oakland Community College, Michigan, USA; 3 Ophthalmology, M. S. Ramaiah Medical College, Bangalore, IND

**Keywords:** applications, artificial intelligence, chatbots, healthcare, medical education

## Abstract

Artificial Intelligence (AI) is being used in every aspect of life today. It has found great application in the healthcare sector, with the use of this technology by medical schools all over the globe. AI has found multiple applications in medical fields such as diagnostics, medicine, surgery, oncology, radiology, ophthalmology, medical education, and numerous other medical fields. It has assisted in diagnosing conditions in a much quicker and more efficient manner, and the use of AI chatbots has greatly enhanced the learning process. Despite the benefits that AI applications provide, such as saving precious time for healthcare givers, there are also concerns regarding AI, mainly, ethical, and the fact that they might render the human race unemployed. However, despite these concerns, a lot of innovations are being made using AI applications, which show a very bright prospect for this technology. Although humans use AI in every part of their daily lives, they are also opposed to its use because they believe it could eventually replace them in the future. In this review of literature, a detailed analysis of the use of AI in the healthcare industry and medical education will be done, along with its shortcomings as well as its future prospects.

## Introduction and background

Artificial intelligence (AI) is defined as the "study of 'intelligent agents', that is, any agent or device that can perceive and understand its surroundings and accordingly take appropriate action to maximize its chances of achieving its objectives." It also describes scenarios in which machines can mimic human minds to learn and analyze and, as a result, solve problems. This is also referred to as machine learning (ML) [1]. AI was first invented and used in 1955 when physicians made the first endeavor to improve their diagnosis using machine and computer-aided programming [2]. During the initial stages, AI was largely limited to academic learning, with scholars investigating the basic ideas of pattern recognition and ML. Over the next couple of decades, it found its place in the medical domain to assist with diagnostic decision-making. However, the intricacies of the medical data and the computing power at the time placed restrictions on these systems [3].

In the 1980s, with the dawn of new systems that began to emulate human expertise, AI has found application in healthcare sectors as well. They helped medical practitioners make important decisions by supporting the interpretation of diagnostic data and medical imagery [4,5]. More advancements were made in the 1990s when machine learning algorithms that could change and get better over time emerged. This was a turning point for AI in medicine because it made it possible for computers to learn from vast amounts of medical data and identify complex links and patterns [6,7]. Nowadays, AI finds its application in a variety of fields such as finance, business, and science, and now its potential is being explored in healthcare sectors, which include both healthcare services and medical education [8]. AI has become an essential component of healthcare education in recent years and has been embraced by numerous medical schools across the globe. Compared to developing nations, the application of AI in medical education is more widespread in Western nations [9].

It is a known fact that AI has changed and will continue to impact every aspect of life in the coming decades, especially in healthcare and medical education. It will revolutionize the practice of medicine in the healthcare industry. However, its implications would probably be felt differently across various disciplines of medicine [10]. During the past decade, there has also been a significant rise in the use of AI for research and development in medical education [11]. However, despite the healthcare industry’s considerable investment in AI technology, the adoption of AI technologies and their implementation in healthcare remains in its infancy. If AI systems are adopted, it would increase the quality of patient care as well as education [12].

In this review of the literature, a detailed analysis of the use of AI in the healthcare industry and medical education will be done, along with its shortcomings and future prospects.

## Review

Application of AI in healthcare services

The AI applications that are used in healthcare are referred to as “medical technology,” which enables health professionals to improve the quality of life for patients and society by completing early diagnosis, minimizing complications, maximizing therapy, and cutting down on hospital stays [13]. The AI software’s modus operandi lies in mimicking the human cognitive functions [14]. AI is present in all aspects of our lives, which we sometimes do not comprehend. For example, Google knows what symptoms and ailments people are looking for; Amazon knows what people want to buy, when, and where; and Netflix knows what movies and series people prefer to watch. These are algorithms that predict these models, which are enabled by data and machine learning. This data is utilized for predicting the healthcare needs of that individual. There is a lot of hope that the use of AI will lead to significant advancements in every aspect of healthcare, from diagnosis to treatment to patient management [7].

They say, “You cannot teach an old dog new tricks.” Healthcare professionals exhibit less familiarity with AI-based applications as evidenced by a web-based questionnaire evaluating medical doctors' perception of AI. Therefore, there is a need to implement specialized education and training programs to enable healthcare professionals to improve patient outcomes using AI applications [15]. Another cross-sectional study conducted on the perception of AI among newly graduated medicine interns reported that more than 50% of the participants were not familiar with the use of AI as a diagnostic application. This highlighted a gap in training toward AI preparedness and a lack of knowledge and know-how regarding the use of AI as a diagnostic tool [16]. This review will highlight how AI can be used in the healthcare industry, including diagnostic applications and medical education.

Application of AI in medicine

**Figure 1:**
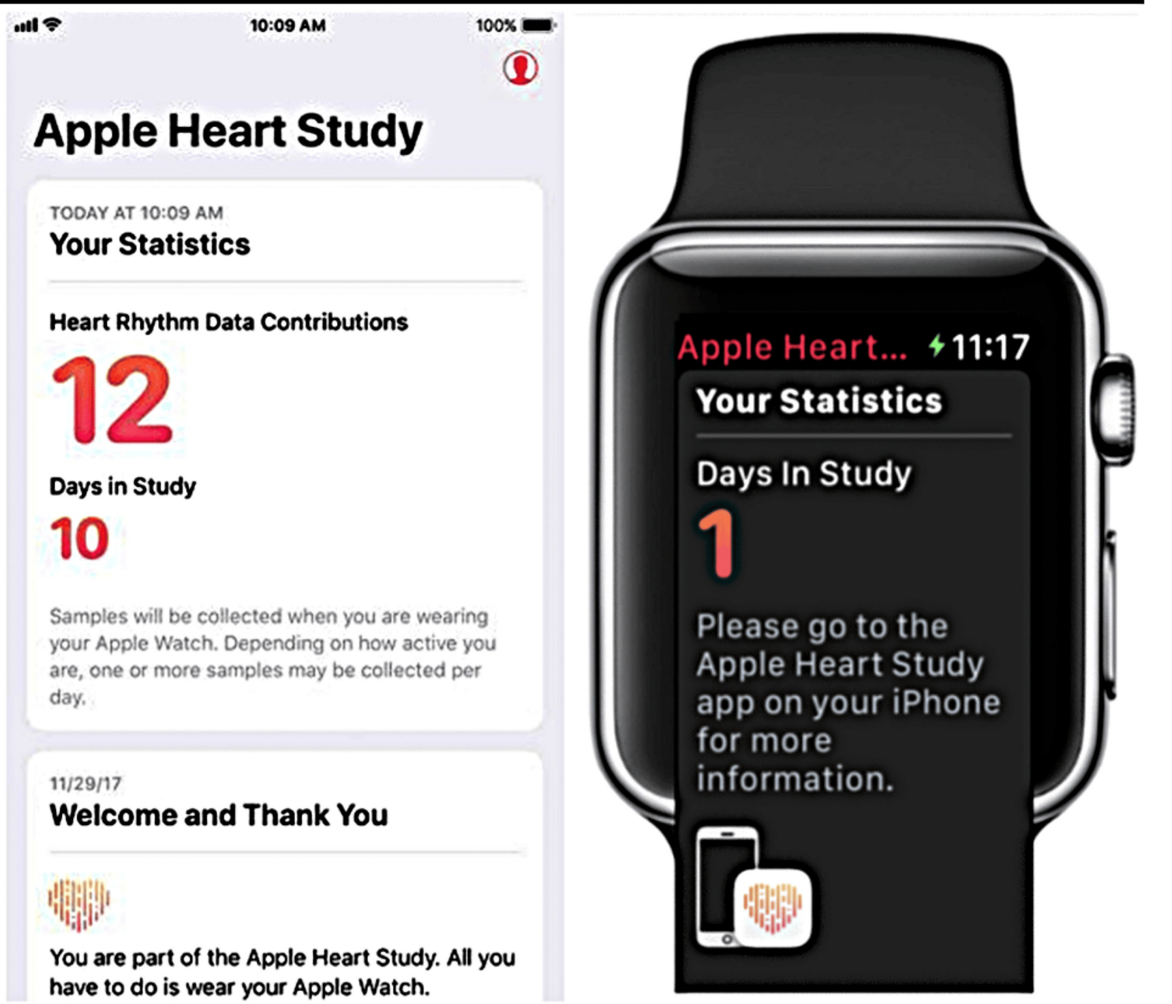
Data display of patients who were part of the Apple Heart Study Source: Turakhia MP et.al. (2019) [19]; used with permission

An AI-based application study consisting of thematic analysis and narrative review was performed during COVID-19. The application utilized machine-based learning algorithms to predict disease patterns and the epidemiology of COVID-19 [17]. The detection of atrial fibrillation (AF) was one of the earliest applications of AI in the healthcare field. According to the study, Assessment of Remote Heart Rhythm Sampling Using the AliveCor Heart Monitor to Screen for Atrial Fibrillation (REHEARSE-AF), mobile patients' remote ECG monitoring with Kardia (Mountain View, California, US) had a higher chance of detecting AF than standard care [18]. Another commonly used feature provided by the Apple Watch (Apple, Los Altos, CA, US), which sought permission from the FDA (Food and Drug Administration), allows easy acquisition of electrocardiogram (EGC), and the patterns and data set can be shared with the healthcare practitioner over a smartphone, thus enabling the practitioner to monitor the patient regularly [19]. The study participants who were in The Apple Heart Study can view the number of days they have been part of the study and the data recorded. This has been shown in Figure 1 [19].

Pulmonary medicine is another field that is a promising avenue for the use of AI. According to a recent study on the subject of interpreting pulmonary function test results, AI-based software offers more precise interpretation and acts as a decision-support tool [20]. Diabetes mellitus, where patients require continuous glucose monitoring, has been made easier by Medtronic (Dublin, Ireland), an FDA-approved manufacturer of AI-based applications. It regulates glucose monitoring, which is smartphone-paired [21]. AI applications have also been utilized in nephrology for the prediction of the decline of glomerular filtration rate (GFR) in patients with polycystic kidney disease and for calculating the risk associated with progressive immunoglobulin A (IgA) nephropathy [22].

In the field of neurology, Empatica (Boston, Massachusetts, US), a medical device manufacturing company, has FDA approval for their wearable device Embrace, which is an intelligent seizure detection device. It detects generalized epilepsy seizures and sends the notification through a mobile application to the physician and the patient’s close confidants about the patient’s location [23]. Stroke, a fairly common condition caused by a thrombus in the blood vessels, has also found a prediction model through AI. It utilizes a movement-detecting device for early stroke prediction. As soon as the patient's movement deviates noticeably from the typical pattern, a stroke alarm is triggered, and therapy is assessed right away. Another wearable device was also proposed, which utilized normal/pathological gaits for the prediction of stroke. These algorithms had up to 90% accuracy for stroke prediction [14].

Application of AI in surgical fields

The use of AI through robotic surgeries has also been on the rise. A structured literature review reported that robotic-assisted surgery had gained popularity in various medical fields such as urology, colorectal, orthopedic, cardiothoracic, and maxillofacial surgeries [24]. Despite being in their infancy and largely managed by humans, surgical robots have already demonstrated encouraging results. As surgical robots can precisely regulate the trajectory, depth, and speed of their actions, they have a hugely positive direct impact on healthcare. In several cases, conducting surgery with a robotic instrument proved to be more efficient than doing it by hand. As the robots never get weary, they are especially well-suited to tasks requiring repetitive, repetitive movements [11].

AI methods for diagnostics have the potential to improve surgery and rehabilitation therapy. Many robots have been created to assist and oversee these kinds of duties. For instance, during motor therapy, rehabilitation robots physically support and guide a patient's limb [2]. In the field of obstetrics and gynecology, AI has substantial potential to improve mother and newborn outcomes and improve clinical decision-making. A systematic review reported that AI models, such as the Adana System (Adana Technologies, Bethesda, MD, US) and Categorical Boosting (Yandex, Moscow, Russia) demonstrated improved prediction accuracy and aided clinical judgment. It was also concluded in the review that AI models are a powerful tool for obstetric care since they anticipate the mode of delivery much better than conventional statistical techniques [25].

Application of AI in radiodiagnosis

ML-based AI has led to significant advancements in healthcare technology, particularly in the field of radiomics. Radiomics utilizes descriptive and predictive models that are based on multimodal medical imaging such as CT and MRI. These models are incorporated with AI applications and utilized for establishing diagnosis [26]. One of the major applications used is cardiovascular magnetic resonance (CMR), which has revolutionized cardiac care through AI. With the use of AI in perfusion mapping, myocardial blood flow may be quickly measured using CMR imaging, providing useful prognostic data for people with suspected cardiovascular disease. Another prominent use of AI is in image analysis, where it analyses MRIs, CT scans, ultrasounds, and X-rays to increase diagnostic precision. For instance, when compared to human radiologists, AI's deep learning (DL) algorithms have shown greater sensitivity and specificity in detecting pneumonia from chest radiographs. AI also aids in automating time-consuming but standard radiology operations, including image interpretation, reporting, scheduling, and invoicing, freeing up medical staff to concentrate on patient care [27,28]. AI programs that have been trained on big collections of medical scan data assist in cutting down on the time and expense involved in image analysis [27].

Application of AI in oncology

**Figure 2:**
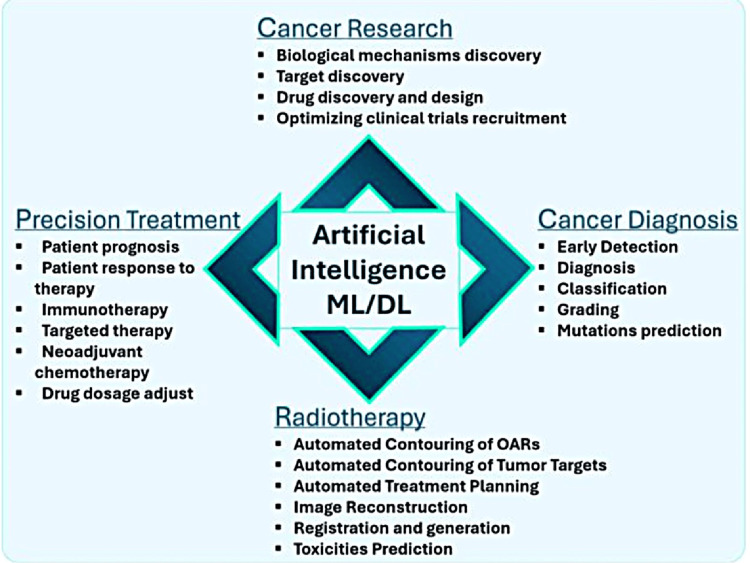
Application of AI across various cancer research and therapeutic fields ML, Machine Learning; DL, Deep Learning; OAR, Organs At Risk; AI, Artificial Intelligence Source: Khanam M et.al. (2024) [27]; reproduced with permission

Over the past decade, AI applications have made significant strides in oncology. With applications including genetic profiling, early cancer diagnosis, tumor classification, grading, forecasting patient outcomes, and treatment responses, AI has achieved tremendous advancements in oncology by utilizing vast datasets and sophisticated computational techniques. AI has the potential to completely transform cancer diagnosis and therapy, ushering in a new era of AI-powered cancer care [27,28]. An example of an AI device application is AI-powered ultrasound, which has a high capability of classifying breast cancer with high precision [27]. Furthermore, AI has also been used to identify potentially dangerous tumors in radiographic pictures that the human eye would miss [14]. It also has its application in neuro-oncology by offering more precise treatment and diagnostic solutions for conditions like brain tumors [29]. Another application of AI has been made in the computational diagnosis of cancer in histopathology, which is capable of diagnosing cancer with high precision [30]. In addition to this, there are numerous applications of AI in cancer treatment, the essence of which is provided in Figure 2 [27].

Application of AI in ophthalmology

Since image recognition is frequently used extensively in the diagnosis and therapeutic monitoring of eye illnesses, the application of AI in ophthalmology is quite promising. AI systems in ophthalmology have demonstrated the efficiency of diagnosing and grading conditions such as diabetic retinopathy at par with or even better than that of skilled ophthalmologists [31]. AI applications have the ability to lower healthcare costs and increase patient admissions for screening and clinical diagnosis in the field of ophthalmology. Also, AI-based DL and algorithms using optical coherence tomography (OCT) images are used for image analytics in ophthalmology [32]. AI is also being utilized for detecting visual field defects, thereby improving the detection of ocular diseases, which are usually missed or are not reported. Despite being relatively new to the area of ophthalmology, AI technologies are rapidly developing and have a big influence on clinical practice and scientific research in the field of ophthalmology [31].

In addition to the detection of diseases, AI also has applications in performing surgeries in ophthalmology. Thereby, robotics has also found its application in this field. The eyes are very delicate; hence, the surgeons' hands need to be exceptionally stable. The preliminary AI application using robotics in ophthalmology has promising results in the scenario of removing membranes from the patient’s eyes or blood beneath the retina caused by age-related macular degeneration. An example of healthcare data-driven AI technology is MedEye, which is being used to accomplish tasks more efficiently and precisely [11].

Application of AI in medical education

Medical education is defined as “a lifetime learning process stretching from undergraduate to postgraduate and specialty training and beyond [11].” AI application in medical education has greatly enhanced since the past decade and more so since the advent of COVID-19, resulting in a global shift to online courses and conferences [33]. It is being used in the student learning process in three ways: direct teaching, support teaching, and empowering the learner. Direct teaching is the methodology in which a teacher directly imparts knowledge to the student. Support teaching is where teachers play a supporting role and collaborate with students as they learn while empowering the learner, where several students can work together to resolve a challenging issue after the teacher provides feedback. Students' knowledge, skill development, and comprehension of intricate medical topics can all be improved by incorporating AI tools into their education [9].

The types of AI used in medical education consist of machine learning (ML), natural language processing (NLP), deep learning (DL), and generative AI such as ChatGPT (Chat Generative Pre-Trained Transformer, OpenAI, San Francisco, CA, US) [14,33]. ChatGPT is an OpenAI tool developed in November 2022 and is one of the most common AI applications used by students [34]. It is also an AI chatbot that is being increasingly used in healthcare systems and education to alleviate the load on healthcare systems [35]. In a study conducted by Gilson et al., it was noted that ChatGPT attained scores comparable to third-year medical students in the United States [36]. In another study, a model was trained using a specific code to answer the questions accurately. This was done to check the proficiency of ChatGPT in answering questions correctly for the Saudi Medical Licensing Exam (SMLE), where ChatGPT achieved an accuracy of 88.6% [37]. Therefore, these studies suggest that ChatGPT can be a useful tool for medical education that enhances learning [36].

The American Medical Association (AMA) passed its first augmented intelligence policy at its 2018 annual meeting. It supported research that emphasized the use of AI in medical education. For example, graduate and postgraduate students at Stanford University's Centre for AI in Medicine and Imaging work to solve healthcare issues with ML. AI systems have helped physicians save time by supplying them with information as clinical problems arise, eliminating the need for them to review previously learned material or scan irrelevant material. This application is being used at Johns Hopkins University School of Medicine, where informatics have been tailored to what and how students are taught [11]. In the field of scientific wiring, ChatGPT is currently assisting scientists and medical researchers with writing abstracts and articles, conducting literature reviews, summarizing data or information, suggesting titles, references, and structure, conducting language reviews to improve the readability of the text, and even creating a complete paper draft [38].

Another teaching method that has gained prominence using AI is the use of simulation-based training (SBT). These are also called medical simulations that provide interactive digital learning by imitating a real-life process or scenario. Such simulations facilitate active learning and decision-making as the students are provided with real-life simulated scenarios [39]. SBT has been known to enhance the learning experience for healthcare professionals. The types of SBTs include high-fidelity mannequins, standardized patients, and hybrid simulations. SBT offers numerous advantages such as improved skill development, decreased mistakes, and the chance to practice repeatedly without endangering actual patients. A common modality used in hybrid simulations is where a mannequin is used as an interactive tool to simulate a patient who is having a medical emergency. [40]. Resusci Anne is a mannequin that was designed in the 1960s for practicing cardiopulmonary resuscitation (CPR) [41]. Nowadays, there are high-fidelity mannequins by Laerdal (Stavanger, Norway) are available that can simulate heart rhythm, breathing, and even complex medical emergencies such as myocardial infarction, angina, acute asthmatic attack, poisoning, etc. These simulators' advanced software enables teachers to regulate and create dynamic and realistic training settings [40].

**Figure 3:**
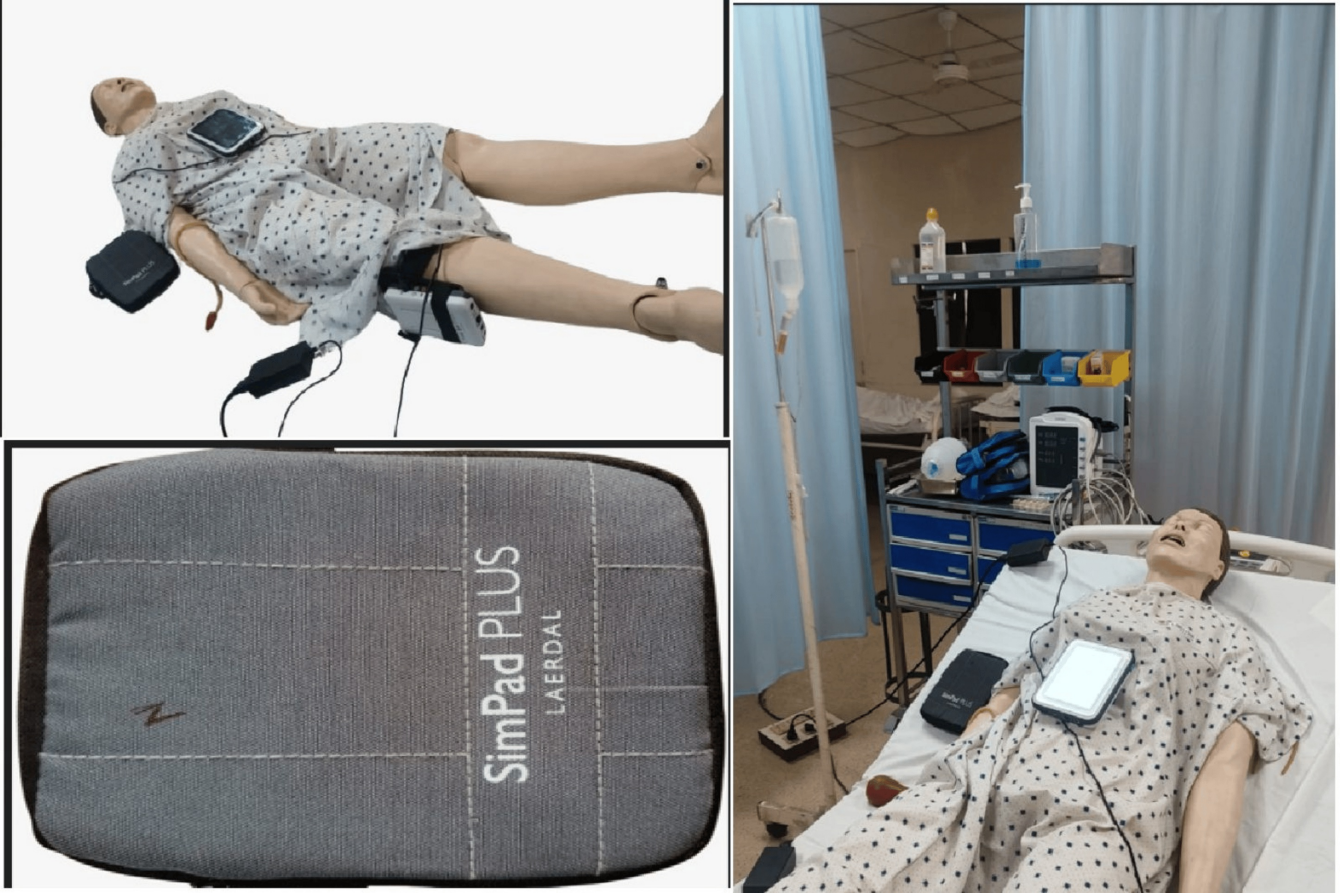
Different modes of SBT used in teaching at Christian Medical College & Hospital, Ludhiana, Punjab, India SBT, Simulation-Based Teaching The Resusci Anne mannequin is used to provide a simulation of medical emergencies connected through SimPAD PLUS, which changes the vitals of the mannequin according to the case scenario. Source: From Central Skills Laboratory at Christian Medical College & Hospital, Ludhiana, Punjab, India. Permission sought from the in-charge of the Central Skills Laboratory.

Christian Medical College & Hospital, Ludhiana, India, has a Central Skills Lab where SimPad Plus, Laerdal is used, which is connected to the Resusci Anne Simulator (Laerdal) to facilitate students in the management of different emergency scenarios. Organophosphorus (OP) poisoning, which is fairly common in the farmlands surrounding the state of Punjab, the clinical presentation of OP poisoning is being simulated on Resusci Anne, which is an AI-based mannequin and is being used as a teaching method for students. The students can interact with the AI-based mannequin through SimPad Plus, Laerdal. The vitals of the patient are adjusted according to what is seen in organophosphorus poisoning and when the appropriate drug is given, the vitals are adjusted to the normal range. As a result, students develop a clear mental image of what occurs during OP poisoning and how it is managed. The models used for SBT in Christian Medical College & Hospital are shown in Figure 3.

Challenges and limitations

There are AI-based watches that record ECG findings and send data to the physician. However, the offset has high false-positive rates due to artifacts and excessive movement. Moreover, elderly people who are prone to cardiac issues do not use this, as it is a major technological barrier for them to overcome [13]. Robotics, which have found a huge application in surgical fields also throws up numerous challenges. The major challenge with these robots is malfunctioning. There is also a high chance of human error or mechanical failure. It is important to comprehend that a single error can be fatal for the patient. Another concern is the cost and the maintenance of the robots, which poses a huge challenge to the implementation of robotics worldwide. It is also a matter of concern on how to procure trained healthcare professionals trained in robotics, as it is a very expensive branch, and doctors with adequate training are few and far between [11].

Ethical concerns have always followed AI since its inception. In the healthcare sector, the main concern is accountability, as any errors in decision-making carry heavy consequences. Since the healthcare professionals did not create or oversee the algorithm in any way, it could seem unjust to hold them responsible while holding the developer responsible would not appear correct as they do not have a say in the clinical decision-making. Therefore, to counter this, in some countries, AI cannot legally make any healthcare decisions without human involvement, and those who do so will be held responsible [12]. Another major concern encountered is consent by the patient. Such a scenario happened in 2018 when Deepmind, an AI-based research laboratory, was acquired by Google (Mountain View, CA, US). It was discovered that the National Health Service (NHS) had provided DeepMind servers with the data of 1.6 million patients without their knowledge to train its algorithm. Their application, Streams, which had an algorithm for managing patients with acute kidney injury, came under fire for collecting data without consent and hence was considered a data breach [12].

There are bright prospects for AI in medical education. However, there are drawbacks to its adoption in various medical educational institutes. The major concern is that both ML and DL require enormous data sets to enable complex but accurate algorithms. However, organizational opposition, data privacy, and security concerns make it difficult for medical institutes to share data to create massive databases. Another concern among teachers in institutes is that they will be replaced by AI-based tools; thereby, there is a lack of acceptability toward the use of AI applications within the institutes [9].

The advent of AI chatbots has revolutionized healthcare service delivery in multiple ways. However, they have certain limitations. The major limitation is that an AI tool like ChatGPT cannot replace the years of experience of a medical professional, nor can it demonstrate empathy, which can only be provided by a human. It is important to note that AI chatbots can only use pre-set data and algorithms; the caliber of the data they receive determines how well they can make recommendations, and any biased or substandard data would give incorrect results [35]. Furthermore, there is the concern of lack of originality, as students are asking similar questions from the same source and getting the same answers, thereby losing their capacity for original thought and becoming incapable of offering convincing arguments in support of their opinions. Although ChatGPT can be used for scientific research and paper writing, the content remains the same, and there are no fresh, innovative ideas. Ultimately, ChatGPT, whose responses depend on training data till it has been trained, will create papers, but it may lack clinical reasoning and thinking [34].

Future directions

AI applications have recently caused a huge uproar in the healthcare industry, even igniting a heated debate about whether AI physicians would someday take the place of human doctors. Although machines will never completely replace human doctors, AI can assist physicians make better clinical decisions or even take the role of human judgment in some functional areas of healthcare [14]. To increase clinical trust and applicability, future research should concentrate on standardizing data collecting, enhancing model interpretability, addressing ethical issues, and guaranteeing fairness in AI predictions [25].

It is imperative for educators to make innovations that engage the learners of the digital era, considering their short attention spans. This generation of students prefers to collaborate in groups and use various software programs to discuss the specifics of their work with other students. They want instant feedback on their work and require individual intellectual support. AI tools have the ability to enhance and address the learning methods of the current generation [11]. In the field of medical education, AI can be used for curriculum development, learning, analysis, and assessment. AI can handle multifaceted problems, increase classification accuracy, reduce the amount of time needed to analyze different curricula, and show how the parameters in curriculum assessment relate to one another. AI can also help students learn by providing them with personalized, adaptable content that is improved by student input. This enables students to identify their knowledge gaps and learn accordingly. Furthermore, AI can effectively deliver thorough, personalized comments and improve the accuracy, speed, and cost-effectiveness of the evaluation process [33].

Facilitators can use a virtual inquiry system as a beneficial analysis tool to understand the requirements of their students. Not every student is the same, and this would help customize the curriculum according to the needs of the student. Teaching them using engaging examples can help them a lot in diagnosing serious diseases [11]. In the field of research writing, ChatGPT has the potential to transform the process by saving time and accelerating the process [42]. So, in the future, AI applications, in general, may be trained to automatically extract and comprehend all pertinent information from electronic health records and by analyzing patient data, including vital signs, lab results, and medical history. This would enable doctors to access patient information more quickly, make recommendations for interventions, and make more thorough and timely decisions [38]. To conclude, AI has the potential to enhance patient engagement, streamline claims management, increase diagnostic accuracy, personalize healthcare delivery, and optimize resource utilization in hospitals [43].

## Conclusions

AI has emerged as a transformative force in healthcare services and medical education, offering significant advancements across various domains, including diagnostics, treatment planning, and personalized care. Despite its promising potential, several challenges and limitations persist. Ethical concerns, accountability, data privacy, high costs, and limited technological proficiency among healthcare professionals further hinder its full integration. Additionally, AI tools like ChatGPT, while valuable in medical education and research, face criticism for their lack of originality, clinical reasoning, and empathetic interaction. However, the SBT method implemented in my institute has received positive responses from students and faculty alike, as it has provided more hands-on training to students to manage emergencies.

Looking ahead, AI will continue to play a pivotal role in shaping the future of healthcare and medical education. Collaborative efforts between AI developers, policymakers, and healthcare providers are essential to ensure responsible implementation and maximize AI’s potential in improving patient care, medical education, and overall healthcare outcomes. With continued innovation and strategic adoption, AI is poised to revolutionize healthcare, empowering professionals and improving the quality of life for patients worldwide.

## References

[1] Rong G, Mendez A, Assi EB, Zhao B, Sawan M (2020). Artificial intelligence in healthcare: review and prediction case studies. Eng J.

[2] Secinaro S, Calandra D, Secinaro A, Muthurangu V, Biancone P (2021). The role of artificial intelligence in healthcare: a structured literature review. BMC Med Inform Decis Mak.

[3] Shortliffe EH (2019). Artificial intelligence in medicine: weighing the accomplishments, hype, and promise. Yearb Med Inform.

[4] Amisha Amisha, Malik P, Pathania M, Rathaur VK (2019). Overview of artificial intelligence in medicine. J Family Med Prim Care.

[5] Bajwa J, Munir U, Nori A, Williams B (2021). Artificial intelligence in healthcare: transforming the practice of medicine. Future Healthc J.

[6] Davenport T, Kalakota R (2019). The potential for artificial intelligence in healthcare. Future Healthc J.

[7] Bohr A, Memarzadeh K (2020). The rise of artificial intelligence in healthcare applications. Artificial Intelligence in Healthcare.

[8] Ahmed MI, Spooner B, Isherwood J, Lane M, Orrock E, Dennison A (2023). A systematic review of the barriers to the implementation of artificial intelligence in healthcare. Cureus.

[9] Narayanan S, Ramakrishnan R, Durairaj E, Das A (2023). Artificial intelligence revolutionizing the field of medical education. Cureus.

[10] Bohler F, Aggarwal N, Peters G, Taranikanti V (2024). Future implications of artificial intelligence in medical education. Cureus.

[11] Mir MM, Mir GM, Raina NT (2023). Application of artificial intelligence in medical education: current scenario and future perspectives. J Adv Med Educ Prof.

[12] Aung YY, Wong DC, Ting DS (2021). The promise of artificial intelligence: a review of the opportunities and challenges of artificial intelligence in healthcare. Br Med Bull.

[13] Briganti G, Le Moine O (2020). Artificial intelligence in medicine: today and tomorrow. Front Med (Lausanne).

[14] Jiang F, Jiang Y, Zhi H (2017). Artificial intelligence in healthcare: past, present and future. Stroke Vasc Neurol.

[15] Banerjee A, Sarangi PK, Kumar S (2024). Medical doctors’ perceptions of artificial intelligence (AI) in healthcare. Cureus.

[16] Sanad AH, Alsaegh AS, Abdulla HM (2024). Perceptions of artificial intelligence in medicine among newly graduated interns: a cross-sectional study. Cureus.

[17] Chen J, See KC (2020). Artificial intelligence for COVID-19: rapid review. J Med Internet Res.

[18] Halcox JP, Wareham K, Cardew A, Gilmore M, Barry JP, Phillips C, Gravenor MB (2017). Assessment of remote heart rhythm sampling using the AliveCor heart monitor to screen for atrial fibrillation: the REHEARSE-AF study. Circulation.

[19] Turakhia MP, Desai M, Hedlin H (2019). Rationale and design of a large-scale, app-based study to identify cardiac arrhythmias using a smartwatch: the Apple Heart Study. Am Heart J.

[20] Topalovic M, Das N, Burgel PR (2019). Artificial intelligence outperforms pulmonologists in the interpretation of pulmonary function tests. Eur Respir J.

[21] Christiansen MP, Garg SK, Brazg R (2017). Accuracy of a fourth-generation subcutaneous continuous glucose sensor. Diabetes Technol Ther.

[22] Niel O, Boussard C, Bastard P (2018). Artificial intelligence can predict GFR decline during the course of ADPKD. Am J Kidney Dis.

[23] Regalia G, Onorati F, Lai M, Caborni C, Picard RW (2019). Multimodal wrist-worn devices for seizure detection and advancing research: focus on the Empatica wristbands. Epilepsy Res.

[24] Connelly TM, Malik Z, Sehgal R, Byrnes G, Coffey JC, Peirce C (2020). The 100 most influential manuscripts in robotic surgery: a bibliometric analysis. J Robot Surg.

[25] Michalitsi K, Metallinou D, Diamanti A, Georgakopoulou VE, Kagkouras I, Tsoukala E, Sarantaki A (2024). Artificial intelligence in predicting the mode of delivery: a systematic review. Cureus.

[26] Mangla S, Garg M, Gill N, Kumar M, Bhatti A, Joseph G, Bhatti N (2024). A review of diagnosing subtypes of breast cancer using MRI radiomics. Afr J Biomed Res.

[27] Khanam M, Akther S, Mizan I (2024). The potential of artificial intelligence in unveiling healthcare’s future. Cureus.

[28] Chen ZH, Lin L, Wu CF, Li CF, Xu RH, Sun Y (2021). Artificial intelligence for assisting cancer diagnosis and treatment in the era of precision medicine. Cancer Commun (Lond).

[29] Surianarayanan C, Lawrence JJ, Chelliah PR, Prakash E, Hewage C (2023). Convergence of artificial intelligence and neuroscience towards the diagnosis of neurological disorders - a scoping review. Sensors (Basel).

[30] Campanella G, Hanna MG, Geneslaw L (2019). Clinical-grade computational pathology using weakly supervised deep learning on whole slide images. Nat Med.

[31] Li Z, Wang L, Wu X (2023). Artificial intelligence in ophthalmology: the path to the real-world clinic. Cell Rep Med.

[32] Anton N, Doroftei B, Curteanu S, Catãlin L, Ilie OD, Târcoveanu F, Bogdănici CM (2022). Comprehensive review on the use of artificial intelligence in ophthalmology and future research directions. Diagnostics (Basel).

[33] Sun L, Yin C, Xu Q, Zhao W (2023). Artificial intelligence for healthcare and medical education: a systematic review. Am J Transl Res.

[34] Jeyaraman M, K SP, Jeyaraman N, Nallakumarasamy A, Yadav S, Bondili SK (2023). ChatGPT in medical education and research: a boon or a bane?. Cureus.

[35] Altamimi I, Altamimi A, Alhumimidi AS, Altamimi A, Temsah MH (2023). Artificial intelligence (AI) chatbots in medicine: a supplement, not a substitute. Cureus.

[36] Gilson A, Safranek CW, Huang T, Socrates V, Chi L, Taylor RA, Chartash D (2023). How does ChatGPT perform on the United States Medical Licensing Examination (USMLE)? The implications of large language models for medical education and knowledge assessment. JMIR Med Educ.

[37] Aljindan FK, Al Qurashi AA, Albalawi IA (2023). ChatGPT conquers the Saudi medical licensing exam: exploring the accuracy of artificial intelligence in medical knowledge assessment and implications for modern medical education. Cureus.

[38] Salvagno M, Taccone FS, Gerli AG (2023). Can artificial intelligence help for scientific writing?. Crit Care.

[39] Dai CP, Ke F (2022). Educational applications of artificial intelligence in simulation-based learning: a systematic mapping review. Comput Educ Artif Intell.

[40] Elendu C, Amaechi DC, Okatta AU, Amaechi EC, Elendu TC, Ezeh CP, Elendu ID (2024). The impact of simulation-based training in medical education: a review. Medicine (Baltimore).

[41] Higham H (2021). Simulation past, present and future-a decade of progress in simulation-based education in the UK. BMJ Simul Technol Enhanc Learn.

[42] Biswas S (2023). ChatGPT and the future of medical writing. Radiology.

[43] Vats K (2024). Navigating the digital landscape: embracing innovation, addressing challenges, and prioritizing patient-centric care. Cureus.

